# Changes in biopsychosocial outcomes for a mixed cohort of ICU survivors

**DOI:** 10.4102/sajp.v74i1.427

**Published:** 2018-04-10

**Authors:** Johannes van Aartsen, Helena van Aswegen

**Affiliations:** 1 Department of Physiotherapy, University of the Witwatersrand, South Africa

## Abstract

**Background:**

Prolonged inflammation and infection associated with being critically ill and the ensuing physical inactivity has proven negative effects on the recovery of physical function, psychological health and reintegration into society for intensive care unit (ICU) survivors. Limited evidence is available on changes in biopsychosocial outcomes for South Africans recovering from an episode of critical illness.

**Objectives:**

To determine changes in biopsychosocial outcomes for a mixed cohort of ICU survivors in hospital and at 1 month and 6 months after discharge.

**Method:**

A prospective, observational, longitudinal study was conducted. Severity of illness, mechanical ventilation (MV) duration and ICU and hospital length of stay (LOS) were recorded. Physical function in ICU test-scored (PFIT-s) was performed at discharge from ICU and hospital. At 1 month and 6 months, peripheral muscle strength, exercise endurance, health-related quality of life (HRQOL), depression status and return to work were assessed. Descriptive and inferential statistics were used.

**Results:**

Participants (*n* = 24) had a median age of 51.5 years, majority were male (*n* = 19; 79%) and most were employed before admission (*n* = 20; 83%). At 6 months, 11 participants (*n* = 11) were part of the final sample. Median PFIT-s changed significantly (0.3 points; *p* = 0.02) between ICU and hospital discharge. Peripheral muscle strength improved significantly for upper and lower limbs over 6 months (*p* = 0.00–0.03) but change in median 6-minute walk test distance (65m) was not significantly different. Significant improvements occurred in mean Medical Outcomes Short Form-36 (SF-36) physical health component scores (8.8 ± 7.6; *p* = 0.00). Mean SF-36 mental health component scores had a strong negative relationship with MV duration (*r* = −0.7; *p* = 0.01), LOS (*r* = −0.56; *p* = 0.04) and Patient Health Questionnaire 9 scores (*r* = −0.72; *p* = 0.01). Six participants (55%) returned to employment.

**Conclusion:**

Clinically important improvements in biopsychosocial outcomes related to physical function and social factors were observed. Limitations in mental aspects of HRQOL were present at 6 months and some reported mild depressive symptoms.

**Clinical implications:**

Intensive care unit survivors with a history of prolonged MV duration and hospital LOS who exhibit limitations in mental HRQOL, and signs of depressive symptoms should be referred to a psychologist for evaluation.

## Introduction

Patients who are acutely ill after major surgery or because of injuries sustained from traumatic events or exacerbation of chronic diseases are admitted to the intensive care unit (ICU) for monitoring and specialised care. There has been a recent shift in approaches to the assessment of quality of care provision in ICU with more emphasis being placed on a patient’s functional abilities, health-related quality of life (HRQOL), psychological health and reintegration into society after discharge instead of mortality or survival rates only (Kayambu, Boots & Paratz 2013).

A recent systematic review and meta-analysis reported that severity of illness on admission to ICU and development of complications associated with critical illness (such as acute respiratory distress syndrome, sepsis or organ dysfunction) have a significant negative impact on long-term outcomes such as physical function, muscle strength and HRQOL of survivors following hospital discharge (Kayambu et al. 2013). This is attributed to the severity of muscle wasting and weakness, fatigue, lethargy that develops as a consequence of prolonged inflammation and sepsis because of critical illness and its ensuing physical inactivity. In some cases, ICU acquired weakness develops (Rattray 2013). It is known that muscle mass decreases at a rate of 2% per day in patients who are sedated or inactive because of acute illness (Farhan et al. 2016). Duration of mechanical ventilation (MV) and ICU and hospital length of stay (LOS) were reported to be associated with poorer muscle strength for upper and lower extremities up to 6 months following discharge in a group of South African survivors of penetrating trunk trauma (Van Aswegen et al. 2010). These authors also reported a significant negative association (*p* = 0.02) between distance walked on the 6-minute walk test (6MWT) and level of morbidity experienced during ICU stay (measured using the Sequential Organ Failure Assessment score) at 6 months after discharge (Van Aswegen et al. 2010).

Psychological problems associated with critical illness may include anxiety, depression, post-traumatic stress, delirium and cognitive dysfunction (Rattray 2013). Up to 28% of ICU survivors present with depression and substantially lower HRQOL than those without depressive symptoms (Davydow et al. 2009). Signs of post-traumatic stress, anxiety and depression are associated with poorer physical health components of HRQOL (McKinley et al. 2016). Results from international studies show that HRQOL of survivors of critical illness is consistently lower than that of general population norms when using standardised outcome measures such as the Medical Outcomes Short Form-36 (SF-36) health survey and the EuroQol Group 5 Dimension (EQ-5D) questionnaires (McKinley et al. 2016; Rattray 2013).

Limited evidence is available regarding the HRQOL of survivors of critical illness within the South African population. A prospective observational study conducted by Van Aswegen et al. (2011) investigated the HRQOL of survivors of penetrating trunk trauma in Johannesburg, reported that participants who survived critical illness showed significant initial reductions in HRQOL. This improved in the first 6 months after discharge for some participants; however, those who required MV for prolonged periods of time experienced more impairments in the physical health component scores (PCS) of HRQOL compared to the mental health component scores (MCS) at 6 months after hospital discharge (Van Aswegen et al. 2011). A cross-sectional study conducted by Schneiderman, Van Aswegen and Becker (2013) investigated the HRQOL of survivors of trauma 6 months after discharge. They showed that these patients experienced reductions in the emotional and physical function domains of HRQOL at 6 months after discharge from hospital. Another South African study by Karachi, Hanekom & Faure (2011), conducted in the Western Cape Province, investigated the survival and HRQOL of 46 patients at 12 months following discharge from an adult surgical ICU. They found that severity of illness measured using the Acute Physiology and Chronic Health Evaluation (APACHE) II scores was correlated to long-term patient survival and the physical functioning domain of HRQOL. Health-related quality of life was also found to be slightly lower in adult patients after surgical ICU admission than that of comparable international ICU populations (Karachi et al. 2011).

Survivors of critical illness may also be faced with challenges when they need to return to their occupation. These challenges may be physical, social, emotional or cognitive in nature. A prospective follow-up study of survivors of critical illness in Norway reported a return to work rate of 55% among study participants, mainly because of post-traumatic stress and lack of optimism (Myhren, Ekeberg & Stokland 2010).

There is a scarcity of literature that describes the biopsychosocial outcomes in relation to the recovery of survivors of critical illness especially in the South African context. Only one study by Van Aswegen et al. (2010) reported objective findings for changes in muscle strength and exercise endurance observed within the first 6 months after discharge. None of the three available studies reported on the level of depression experienced by ICU survivors or their rate of return to work after discharge. For the purpose of this article, biopsychosocial outcomes refer to outcomes that measured physical function (physical function in ICU test-scored [PFIT-s], peripheral muscle strength, exercise endurance and HRQOL related to physical function), psychological health (level of depression and HRQOL related to psychological health) and social factors (social functioning [SF], home environment, type of occupation and return to work rate) related to the recovery of ICU survivors.

## Aims and objectives

The aim of this study was to determine the changes in biopsychosocial outcomes for a mixed cohort of ICU survivors in hospital and over the first 6 months after hospital discharge. Specific objectives were to establish the level of and changes in (a) physical function (PFIT-s, peripheral muscle strength, exercise endurance and HRQOL related to physical function), (b) psychological health (HRQOL related to mental health and level of depression) and (c) social factors (SF, home environment, type of occupation and return to work rate) for survivors of critical illness within the abovementioned time frames.

An additional objective was to determine if any relationship existed between severity of illness on ICU admission (APACHE II and Simplified Acute Physiology Score [SAPS] II); duration of MV, ICU and hospital LOS; HRQOL; physical function; and level of depression.

The null hypothesis used was that there is no significant improvement in recovery of physical function, psychological health and return to work rate within 6 months after discharge in a mixed cohort of South African survivors of critical illness.

## Methodology

This was a prospective, observational, longitudinal study. Participants were recruited from Netcare Union Hospital in Alberton and Netcare Mulbarton Hospital in Mulbarton. Both are situated in South Gauteng Province of South Africa. A consecutive sampling method was used to identify and recruit participants from the ICU of the abovementioned hospitals over a 13-month period. Patients aged 18 years or older of both genders who were admitted to the ICU because of respiratory failure (pneumonia, exacerbation of chronic obstructive pulmonary disease, or penetrating or blunt trauma to the chest wall), organ dysfunction or for postoperative care (exploratory surgery following trunk trauma, elective cardiac surgery, e.g. coronary artery bypass graft [CABG] surgery or valve replacement), and who were on MV for more than 24 h with a Glasgow Coma Scale score of 15 were eligible for inclusion. Patients with terminal illness, cognitive impairments or history of mental disorders, multiple orthopaedic injuries and unstable cardiac conditions (e.g. myocardial infarction if they did not undergo CABG surgery, uncontrolled cardiac arrhythmias, aortic stenosis, pulmonary infarction, myocarditis, pericarditis and dissecting aneurysm) were not considered for participation.

Population size was estimated by reviewing the patient admission registers of the two ICUs over a 4-month period. Approximately 163 patients in total were admitted on a monthly basis to ICU of which an average of 13 patients per month fitted the inclusion criteria for this study. It was therefore estimated that approximately 156 patients would potentially be available for inclusion over a 12-month recruitment period, depending on their rate of survival as a result of critical illness and their willingness to participate. Additionally, a sample size of 163 participants was determined for the study using an online sample size calculator for prevalence studies (Naing, Winn & Nordin 2006). The calculated sample size estimate was based on finite population correction for an 8% prevalence of suitable patients from a population size of 1956 at 4% precision (*d* = 0.04).

## Measuring instruments

Severity of illness was measured by calculating the APACHE II and SAPS II scores. The APACHE II is a general mortality prediction model widely used in the ICU (Badreldin et al. 2011). A freely available online calculator was used to calculate APACHE II scores. The validity and reliability of the APACHE II scoring system is well established (Chou et al. 2010; Grmec & Gasparovic 2001; Raj et al. 2014; Tsai et al. 2012). The SAPS II is used to calculate the probability of hospital mortality for ICU patients over the age of 18 years (Badreldin et al. 2011). A freely available online calculator was used to calculate SAPS II scores for each participant. During the initial development and validation of the SAPS II, it showed good validity for a large international sample (Le Gall et al. 1993). Capuzzo et al. (2000) reported the SAPS II to be valid in the surgical ICU population.

The first author extracted data from all participants’ files and calculated scores (using the respective online calculators) for the APACHE II and SAPS II outcomes to increase the reliability of information obtained.

Peripheral muscle strength was assessed using a MicroFet2^®^ hand-held dynamometer (HHD) (Hoggan Health Industries, Inc., Draper, Utah). Muscle strength was measured in Newtons. The reliability and validity of the HHD is well established in various patient populations (Gontkof et al. 2008; Roy & Doherty 2004; Taylor, Doff & Graham 2004; Van Aswegen et al. 2010).

Exercise endurance was assessed using the 6-minute walk test (6MWT). The 6MWT has excellent short-term reproducibility when administered by the same assessor (ATS2002). The 6MWT is reliable and valid and has been used in several studies on the outcomes of survivors of critical illness (Aitken et al. 2012; Herridge et al. 2003; Van Aswegen et al. 2010).

HRQOL was assessed using the SF-36 and EQ-5D questionnaires. The following translations of the SF-36 questionnaire were used in this study (depending on patient preference): English (South African), Afrikaans, Sesotho and Zulu. Scores for the SF-36 were calculated using the Quality Metrics Health Outcome Scoring Software 4.5. This was provided by Quality Metrics through Optum Insight Life Sciences Inc. together with the licence to administer the SF-36. The SF-36 questionnaire has been comprehensively validated in the critically ill patient population (Dowdy et al. 2005; Khoudri et al. 2012) and is reported to be an acceptable, reliable and valid outcome measure to assess HRQOL in survivors of critical illness (O’Neill et al. 2014). The SF-36 questionnaire utilises 36 items to obtain two summary scores of physical and mental health and eight domain scores of HRQOL (Dowdy et al. 2005). The EQ-5D questionnaire is a simple preference-based health profile measure of HRQOL of which the reliability and validity have been well established for survivors of critical illness (Flaatten et al. 2008; Khoudri et al. 2012). The EQ-5D was available to participants in the following language translations (depending on patient preference): English (South African), Afrikaans, Zulu, Sesotho, Setswana, and Xhosa. Permission to use the EQ-5D was provided by the EuroQol Group Foundation. The EQ-5D consists of two sections: the first section consists of five questions (each with three possible responses) that evaluate various dimensions (mobility, self-care, usual activities, pain or discomfort, and depression or anxiety) of HRQOL; the second section consists of a visual analogue scale (VAS) in the form of a vertical line that depicts worst to best possible health state (Chen, McGhee & Pang 2014).

The presence and severity of depression symptoms among study participants was screened by using the Patient Health Questionnaire 9 (PHQ-9). The PHQ-9 is a reliable and valid self-administered, easy-to-use screening tool commonly used for detecting depression (Wu 2014). It consists of nine self-report questions about symptoms of depression. A score of 8 to 11 on the PHQ-9 is recommended as the cut-off score for depression (Kumei, Nozu & Ohhira 2015). The PHQ-9 score indicates the presence of mild, moderate, moderately severe and severe depression. It is free for usage to online users and is available in more than 35 languages, including English. There are no South African translations of the PHQ-9 questionnaire. In this study, the PHQ-9 was available to participants in English only.

Physical function of participants in ICU was assessed using the PFIT-s. The PFIT-s is a reliable, valid and responsive measure used in ICU patient populations to assess physical function (Denehy et al. 2013; Skinner et al. 2009).

## Procedure

Prior to commencement of recruitment and data collection for the study, a pilot study was performed to familiarise the first author and research assistant, a physiotherapist, with a BSc (Physiotherapy) and extensive experience in critical care, with all the outcome measures to be used. Intra-rater reliability for PFIT-s, HHD and 6MWT between the first author and research assistant was assessed using eight patients recruited from the ICUs mentioned above. Pearson’s correlations were calculated between different measurements to calculate intra-rater reliability. Strong positive correlations were found between the first author and research assistant for measurements for the PFIT-s (*r* = 1; *p* = 0.00), right-sided knee flexion HHD (*r* = 0.88; *p* = 0.02), right-sided elbow extension HHD (*r* = 0.99; *p* = 0.00) and 6MWT distance (*r* = 0.99; *p* = 0.00). The intra-rater reliability was shown to be sufficient for these measures. The mean duration of time it took participants to complete all four questionnaires was 20 min.

Patients admitted to the ICUs of the two mentioned research sites were screened daily by the first author or research assistant for eligibility for the study. The first author or assistant approached patients who fitted the inclusion criteria, explained the nature of the study, using a study information sheet, on the day prior to discharge from the ICU to the ward and obtained written consent. Only patients with a Glasgow Coma Scale score of 15 with the ability to understand and respond appropriately to simple questions were approached. After consent was obtained, the first author captured information for the demographic questionnaire (name, age, gender, contact details, previous disability or pre-morbid conditions, living arrangements, highest level of education and type of occupation) during a short interview.

The first author captured information needed to calculate APACHE II and SAPS II scores from individual participants’ ICU charts along with the duration of MV and ICU LOS. Within 24 h after ICU discharge, the PFIT-s was assessed by the patient’s bedside in the ward. All participants received physiotherapy treatment for the duration of their hospital stay. Prior to hospital discharge, PFIT-s was assessed again by the first author or assistant. Hospital LOS was recorded at the time of discharge.

Each participant was sent a mobile text message (1 week before the 1-month and 6-month follow-up appointments, respectively) and was phoned (2 days before the appointment) as a reminder and confirmation of their attendance.

At the 1-month follow-up, demographic information was verified with each participant (including details on return to employment). The participant then performed the 6MWT according to the American Thoracic Society (ATS) procedure (2002), with the only modification being that a 10-m track was used. This modification was necessary as limited space was available inside the treatment rooms where participants were assessed as weather conditions were variable outdoors. The distance was demarcated by cones, and participants were instructed to walk around the cones as they returned to the starting point. This ensured less time lost during the test related to stopping and turning around and a more continuous walking pace was obtained, although a longer test distance would have been preferred.

Blood pressure, heart rate, respiratory rate, peripheral oxygen saturation and distance walked were recorded. The first 6MWT was performed to allow the participant to familiarise himself or herself with the testing procedure. The 6MWT was repeated after a 30-min rest period. The better result of the two tests was captured for data analysis.

After the 6MWT assessment was completed, the participant was asked to complete the SF-36, EQ-5D and PHQ-9 questionnaires. The questionnaires were provided in each participant’s preferred language except for the PHQ-9. The first author explained the purpose of each questionnaire to the participant and provided instructions on how to complete it according to the prescribed guidelines. All participants were literate and were able to complete all questionnaires.

Peripheral muscle strength testing followed. The procedures performed and muscle groups tested are summarised in Table 1. A practice trial was performed with each participant prior to measurements being recorded to ensure they understood the testing procedure and experienced the feel of pushing against the dynamometer. The first author explained each movement or muscle group tested to the participant prior to testing. During the tests, isometric force was measured using ‘make’ tests. The participant was asked to build their force gradually over 2 s and then maintain a maximal contraction for 5 s. During this 7-s procedure, the first author held the dynamometer stationary against the limb segment involved (Andrews, Thomas & Bohannon 1996).

**TABLE 1:** Peripheral muscle strength testing positions and muscle groups tested.

Muscle action tested (muscle group tested)	Patient position Joint position	Dynamometer placement	Stabilisation region
Shoulder flexion (anterior deltoid)	Supine Shoulder flexed to 90° and elbow extended	Slightly proximal to humeral epicondyles	Axillary region
Elbow flexion (biceps brachii)	Supine Elbow flexed to 90° with the forearm supinated	Slightly proximal to styloid process of the radius	Superior surface of the arm or shoulder
Elbow extension (triceps brachii)	Supine Elbow flexed to 90° with the forearm in neutral	Slightly proximal to the ulnar styloid process	Anterior surface of the arm or shoulder
Hip abduction (gluteus medius)	Supine Both legs in neutral	At the point of the lateral femoral condyles	Stabilisation of the opposite leg in a neutral position
Knee flexion (hamstrings)	Sitting at the edge of the bed Knees and hips flexed to 90°; participant’s hands resting on his or her lap	Slightly proximal to the malleoli on the posterior aspect of the lower leg	An assistant stabilises at the shoulders
Knee extension (quadriceps femoris)	Sitting at the edge of the bed Knees and hips flexed to 90°; participant’s hands resting on his or her lap	Slightly proximal to the malleoli on the anterior aspect of the lower leg	An assistant stabilises at the shoulders
Ankle dorsiflexion (tibialis anterior)	Supine The hip, knee and ankle at 0°	Slightly proximal to the metatarsophalangeal joints of the foot	Knee to be kept in full extension, leg supported on the bed while the foot is off the bed

The procedures for the 6-month follow-up were exactly the same as described for the 1-month follow-up above.

Throughout all assessment sessions, emergency medical equipment was available for use. The first author and assistant were trained in basic life support and in the use of an automated external defibrillator. No emergency situations arose during the data collection period.

Data captured were ordinal, interval and ratio in nature. The Statistical Programming for the Social Sciences Statistics (SPSS) Version 23 software package was used for data analysis. Descriptive statistics were used to present the data. Data were normally distributed. Continuous data (age, PFIT-s interval scores, 6MWT distance, peripheral muscle strength, APACHE II and SAPS II scores, SF-36 scores, EQ-5D VAS, and LOS) were summarised as means and standard deviations (SD) and median and interquartile range (IQR) because of the small sample size (*n* = 24). Categorical data (gender, type of occupation, diagnosis, comorbidities, and EQ-5D scores) were summarised as frequencies and percentages. Paired samples t-test was used to assess changes in continuous data over time. Chi-square test was used to compare categorical data. Because of the small sample size, Spearman’s rho was used to measure the degree of association between variables. A linear regression model was used to assess relationships between multiple variables. Testing was performed at the 0.05 level of significance (*p* ≤ 0.05) and confidence intervals (CI) were set at 95%.

### Ethical considerations

Permission to perform the study was obtained from the University of the Witwatersrand Human Research Ethics (Medical) committee (certificate number: M130404). Permission to perform the study in hospitals belonging to the Netcare group was obtained from the Netcare Research Ethics committee (certificate number: UNIV-2014-005). Permission was also obtained from the hospital management of Netcare Union and Netcare Mulbarton hospitals to access patients’ ICU charts and files and to recruit patients for this study. For the purpose of patient follow-up after discharge from the acute care setting, permission was obtained from the hospital management of the following rehabilitation facilities: Netcare Rosebank Rehabilitation Centre, Life Kensington Clinic, Netcare Rehabilitation Hospital and Life Riverfield Lodge. This study was registered on the South African National Clinical Trials Registry (trial number DOH-27-0614-4751). Participants were provided with information about the study, and written consent was obtained from those who agreed to participate.

## Results

**FIGURE 1:**
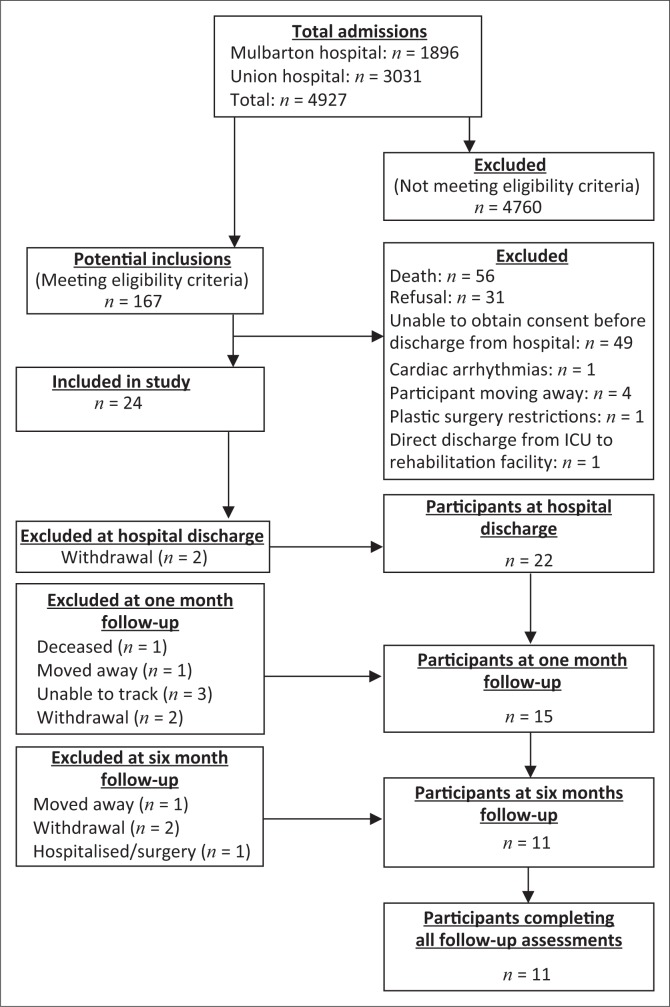
Flow of participations throughout the study.

A total of 4927 patients were admitted to the ICUs of the two participating hospitals during the study period. Participants’ flow through the study is summarised in Figure 1. Twenty-four participants were enrolled into the study. The median age was 51.5 (IQR 41.3–60.8) years, the majority were male (*n* = 19, 79%) and most participants were employed (*n* = 20, 83%) prior to the onset of critical illness. Half of the participants in this study presented with pre-existing disease prior to critical illness (*n* = 12; 50%) (Table 2). No participants reported a smoking history, history of alcohol or substance abuse or a history of dietary abnormalities. The majority of participants underwent surgical interventions (*n* = 19, 79%) which led to ICU admission (Table 3). Median duration of MV was 7 days (IQR 2.3–15.3), median ICU LOS was 15.5 days (IQR 10.3–28) and median hospital LOS after ICU discharge was 4.5 days (IQR 2–8.5). The median APACHE II score was 12.5 (IQR9.3–21.8) and median SAPS II score was 41.5 (IQR 27–64.8).

**TABLE 2:** Baseline characteristics and demographics for study participants.

Characteristics	*N*	Percentage (*n* = 24)
**Gender** Male Female	19 5	79.2 20.8
**Handedness** Right Left	24 0	100 0
**Living arrangements** Living alone Living with others Support available at home	3 21 24	12.5 87.5 100.0
**Level of education** Grade 9 Grade 11 Grade 12 Diploma Basic degree qualification Honours degree qualification Master’s degree qualification	1 1 9 7 2 2 2	4.2 4.2 37.5 29.2 8.3 8.3 8.3
**Type of occupation** Unemployed Physical labourer Office worker Pensioner	1 5 15 3	4.2 20.8 62.5 12.5
**Previous disabilities and pre-morbid diseases** Dyslipidaemia Hypertension Cardiac stenting surgery Cardiac valve replacement surgery Diabetes mellitus Chronic renal failure Laparotomy Visual difficulties Cancer Low back pain	12 3 1 1 1 1 1 1 1 1 1	50.0 25.0 8.3 8.3 8.3 8.3 8.3 8.3 8.3 8.3 8.3

**TABLE 3:** Reasons for admission and types of surgery recorded for study participants.

Admission type	*N*	Percentage (*n* = 24)
**Medical admissions** Pneumonia Pulmonary tuberculosis Pleural effusion Pulmonary oedema Chronic obstructive pulmonary disease (COPD) Septicaemia Chronic renal failure	8 2 1 1 1 1 1 1	33.3 8.3 4.2 4.2 4.2 4.2 4.2 4.2
**Trauma admissions** Penetrating thorax trauma Blunt trauma to the thorax and abdomen	7 1 6	29.2 4.2 25.0
**Cardiac admissions** Congestive cardiac failure Coronary artery bypass graft Mitral valve replacement Aortic valve replacement, mitral valve replacement and coronary artery bypass graft	7 1 4 1 1	29.2 4.2 16.7 4.2 4.2
**Surgical interventions** Elective surgical intervention Non-elective surgical intervention	19 6 11	70.8 25.0 45.8

### Physical function

The median PFIT-s interval score was 6.8 (IQR 6–7.7) at ICU discharge and 7.1 (IQR 6.4–7.9) at hospital discharge. A significant change of 0.3 points between PFIT-s interval scores at ICU discharge and hospital discharge was found (*p* = 0.02). No significant relationship was found between PFIT-s, APACHE II and SAPS II scores, duration of MV, ICU LOS or hospital LOS. Significant changes in the mean difference in peripheral muscle strength at 6 months after discharge were observed for right-sided elbow flexion (17.3 ± 15.7, 95%CI: −27.8 to −6.8, *p* = 0.00), elbow extension (32.6 ± 37, 95%CI: −57.5 to −7.7, *p* = 0.02), hip abduction (53.4 ± 52.3, 95%CI: −88.5 to −18.2, *p* = 0.01), knee extension (59.9 ± 40.3, 95%CI: −87 to −32.8, *p* = 0.00), knee flexion (42.4 ± 31.3, 95%CI: −63.4 to −21.4, *p* = 0.00) and ankle dorsiflexion (29.9 ± 36.9, 95%CI: −54.7 to −5.1, *p* = 0.02), left-sided hip abduction (36.4 ± 45.2, 95%CI: −66.7 to −6, *p* = 0.02), knee flexion (40 ± 43.3, 95%CI: −69 to −10.9, *p* = 0.01) and ankle dorsiflexion (43.3 ± 48.1, 95%CI: −75.7 to −11, *p* = 0.03).

The median distance walked on the 6MWT was 425 m (IQR 332.5–517.5) at 1 month and 490 m (IQR 400–570) at 6 months. The percentage of predicted distance achieved on the 6MWT was calculated using the equation: expected 6MWT distance = 868.8 – (2.99 × age) – (74.7 × gender) (male = 0; female = 1) (Gibbons et al. 2001). Median percentage of predicted distance achieved was 62% (IQR 48.6–76) at 1 month and 73% (IQR 58–83.4) at 6 months. There was a non-significant increase of 65 m in median 6MWT distance achieved (*p* = 0.6). The change in predicted 6MWT distance achieved (11%) was also not significant (*p* = 0.53). A moderate strength, positive relationship (*r* = 0.53; *p* = 0.09) was found between the duration of MV and 6MWT distance and SF-36 PCS and 6MWT distance (*r* = 0.54; *p* = 0.09) at 6 months after discharge but were not significant.

### Psychological health

Four participants (27%) had mild severity of depressive symptoms at 1 month and two participants (18%) had mild depressive symptoms at 6 months after discharge. Only one participant (6.7%) presented with major depressive disorder at 1 month after discharge. The median PHQ-9 score was 3 at 1 month and 1 at 6 months. There was a non-significant change of 2 points during the 6 months period (*p* = 0.22).

Results obtained from the SF-36 questionnaire are presented in Table 4. A significant difference in the mean PCS of 8.8 points (± 7.6, 95%CI: −13.9 to −3.7, *p* = 0.00) was observed over the 6 months period. Significant changes were also observed for the role physical (RP) (*p* = 0.001), bodily pain (BP) (*p* = 0.05), general health (GH) (*p* = 0.001), vitality (VT) (*p* = 0.01) and SF (*p* = 0.001) domains.

**TABLE 4:** Mean SF-36 domain and summary scores for participants obtained at 1 month and 6 months after discharge.

SF-36 Domain	SF-36 scores				
	Mean at 1 month (± SD) (*n* = 15)	Mean at 6 months (± SD) (*n* = 11)	Mean difference (± SD)	95% CI	*p*
Physical component score (PCS)	42.9 (8.8)	51.7 (10.5)	8.8 (7.6)	(−13.9, −3.7)	0.00*
Mental component score (MCS)	49.2 (10.5)	53.3 (7.7)	4.0 (7.7)	(−9.2, 1.1)	0.11
Physical function (PF)	63.2 (16.7)	77.3 (20.8)	14.1 (17.0)	(−25.5, −2.7)	0.21
Role physical (RP)	46.0 (26.3)	80.1 (27.1)	33.5 (24.6)	(−50.0, −17.0)	0.00*
Bodily pain (BP)	59.0 (22.5)	72.2 (28.1)	13.2 (19.4)	(−26.2, −0.1)	0.05*
General health (GH)	70.1 (18.9)	82.0 (16.3)	11.9 (10.8)	(−19.2, −4.6)	0.00*
Vitality (VT)	57.4 (15.5)	77.3 (17.7)	19.9 (21.8)	(−34.5, −5.2)	0.01*
Social functioning (SC)	58.0 (28.7)	84.1 (19.4)	26.1 (27.6)	(−44.7, −7.6)	0.01*
Role emotional (RE)	72.0 (29.6)	84.1 (19.5)	12.1 (25.4)	(−29.2, 4.9)	0.14
Mental health (MH)	76.3 (18.7)	76.8 (17.1)	0.5 (18.6)	(−13.0, 12.1)	0.94

SD, standard deviation; CI, confidence interval.

* , indicates significance at level *p* ≤ 0.05.

The mean EQ-5D VAS score was 72.5 (± 15.9) at 1 month and 87.2 (± 11.1) at 6 months. A significant change of 14.6 points (± 9.7) in mean EQ-5D VAS scores was observed over the 6 months after discharge (*p* = 0.00) (95%CI: −21.2 to −8.1). Table 5 summarises participants’ responses to the five questions in the EQ-5D. Their abilities to mobilise, care for themselves and participate in usual activities improved over the 6-month period. Moderate strength negative relationships, which were statistically significant, were identified between the SF-36 MCS and ICU LOS (*r* = −0.56; *p* = 0.04) and hospital LOS (*r* = −0.56; *p* = 0.04) at 6-month follow-up. A strong negative relationship was found between the SF-36 MCS scores at 6-month follow-up and duration of MV (*r* = −0.7; *p* = 0.01). A strong negative relationship was found between SF-36 MCS scores and PHQ-9 scores at 6 months after discharge from the hospital (*r* = −0.72; *p* = 0.01).

**TABLE 5:** Response frequencies for categorical data obtained for the various domains of the EuroQol Group 5 Dimension.

EQ-5D domains	At 1 month follow-up (*n* = 15) *n* (%)	At 6-month follow-up (*n* = 11) *n* (%)
**Mobility** No problem Problem	9 (60.0%) 6 (40.0%)	10 (90.9%) 1 (9.1%)
**Self-care** No problem Problem	11 (73.3%) 4 (26.7%)	10 (90.9%) 1 (9.1%)
**Usual activities** No problem Problem	6 (40.0%) 9 (60.0%)	9 (81.8%) 2 (18.2%)
**Pain or discomfort** No problem Problem	3 (20.0%) 12 (80.0%)	9 (81.8%) 2 (18.2%)
**Anxiety or depression** No problem Problem	10 (66.7%) 5 (33.3%)	8 (72.7%) 3 (27.3%)

EQ-5D, EuroQol Group 5 Dimension.

### Social factors

The majority of participants (*n* = 21; 87.5%) lived with others prior to admission and many reported that they had support from others at home after hospital discharge. The highest level of education for many participants was Grade 12 schooling (*n* = 9; 37.5%) or diploma after matriculation (*n* = 7; 29.2%) and the majority were office workers (*n* = 15; 62.5%). Of the 11 participants who were assessed at 6 months, 6 participants had returned to employment (55%). Five of the six (83%) participants had returned to their pre-admission employment. Reasons reported by participants for not returning to work are summarised in Table 6. No significant relationship was found between the presence of depressive symptoms and return to work rate.

**TABLE 6:** Reasons for not returning to work at 1 and 6 months.

Reason for not returning to work	One month		Six months	
	*N*	Percentage (*n* = 12)	*N*	Percentage (*n* = 5)
Physical weakness	2	16.7	0	0
Readmission to hospital	1	8.3	0	0
Family pressure†	1	8.3	0	0
Personal preference	2	16.7	0	0
Pensioner status	6	50.0	5	100

† , family pressure: The family of the participant recommended to the participant not to return to work.

## Discussion

This study contributes to the available limited evidence in South Africa on changes in biopsychosocial outcomes of survivors of critical illness in hospital and over the first 6 months after discharge. The majority of participants were males which is similar to the gender-related demographics described by other South African researchers (Schneiderman et al. 2013; Van Aswegen et al. 2011). The participants in this study were older than those described by Schneiderman et al. (2013) and Van Aswegen et al. (2011), and more participants had pre-existing diseases prior to ICU admission. LOS in ICU and hospital was shorter than that described for the polytrauma population by Schneiderman et al. (2013) and the long MV penetrating trunk trauma group in the study by Van Aswegen et al. (2011). Severity of illness (APACHE II) scores for participants in this study were similar to those described for the polytrauma group by Schneiderman et al. (2013) but less than that described for the penetrating trunk trauma group (Van Aswegen et al. 2011).

Physical function in ICU and prior to hospital discharge was assessed using the PFIT test. The 0.3 points change observed in PFIT-s interval scores between ICU and hospital discharge was statistically but not clinically significant. Denehy et al. (2013) reported that the minimal clinically important difference in PFIT-s interval scores for a medical and surgical ICU population is 1.5 points. The PFIT-s has moderate convergent validity with the 6MWT (Denehy et al. 2013). These authors argued that the 6MWT is not a reliable test to use for the assessment of physical function in ICU as a large space is required to perform the test and patient attachments and drains during walking and turning might influence results obtained. In contrast, the PFIT-s requires less space and is easier to perform in ICU (Denehy et al. 2013). A ceiling effect for PFIT-s interval scores at the point of ICU discharge was reported by Denehy et al. (2013) and might explain the small change in scores observed for this cohort at hospital discharge. Assessment of physical function using the 6MWT at hospital discharge might therefore have been more appropriate.

Participants’ ability to perform exercise (6MWT) seemed to improve over the 6 months after discharge but the change in distance walked (65 m) was not statistically significant. Van Aswegen et al. (2010) reported a 74 m change in distance walked with the 6MWT for penetrating trunk trauma participants who underwent prolonged MV duration over the same time period. Elliott et al. (2011) reported a change of 36 m in 6MWT distance achieved at 6 months after discharge for their ICU survivors (control group) who were of similar age and gender distribution to this cohort but had a higher severity of illness. The reported minimum clinically important difference (MCID) for 6MWT in patients with chronic heart failure and for those with chronic obstructive pulmonary disease (COPD) is 30 m (Polkey et al. 2013; Shoemaker et al. 2013). Even though there was less change in distance walked on the 6MWT for the current cohort compared to the penetrating trauma group, the change of 65 m is clinically relevant when compared to that achieved by Australian ICU survivors (Elliott et al. 2011) and those with chronic diseases (Polkey et al. 2013; Shoemaker et al. 2013). Risk for mortality related to chronic disease is low for this cohort because of the great change in distance walked on the 6MWT (Polkey et al. 2013; Shoemaker et al. 2013). A limitation of this study is that respiratory muscle strength was not assessed, so it is not known whether respiratory muscle weakness existed or influenced participants’ performance during the 6MWT.

Significant improvements in muscle strength for both upper and lower extremities were observed for participants over the 6 months period. In their review on the effects of exercise in critically ill patients, Kayambu et al. (2013) reported that exercise and mobilisation in ICU had a significant positive effect on improvement in peripheral muscle strength. The in hospital physiotherapy that all participants in this study received might therefore offer an explanation for the significant improvements in muscle strength observed over time. Van Aswegen et al. (2010) reported that their penetrating trunk trauma participants who had a short duration of MV had a significant improvement in upper and lower extremity muscle strength within the first 3 months following hospital discharge, whereas those who experienced prolonged MV had persistent muscle weakness in their extremities up to 6 months following discharge. They found a strong association between hospital LOS and upper and lower extremity strength (Van Aswegen et al. 2010). A limitation of our study is that associations between muscle strength and LOS and severity of illness were not assessed.

Less improvement in mental HRQOL compared to physical HRQOL was observed for this cohort. A small number of participants presented with mild depressive symptoms at 6 months after hospital discharge. Jackson et al. (2014) studied the presence of depression and post-traumatic stress on mental health in a large cohort of ICU survivors. They reported that up to a third of their participants presented with symptoms of mild depression at 3 months after discharge and that depression was present in those without a history of depression prior to ICU admission (Jackson et al. 2014). McKinley et al. (2016) reported an independent association between the presence of depressive symptoms and mental health summary scores in their group of ICU survivors when assessed at 6 months after discharge. Schneiderman et al. (2013) similarly reported lower MCS scores (58.7) than PCS scores for their polytrauma group at 6 months following discharge. In contrast, Van Aswegen et al. (2011) reported higher MCS scores than PCS scores for their penetrating trunk trauma participants at 6 months. They attributed this finding to the fact that many participants expressed their relief at being alive after the injuries that they sustained. The limited improvement in mental HRQOL for the current cohort might be explained by their older age and the presence of pre-existing illness prior to admission. Somatic symptoms such as lack of energy, sleep disturbance and lack of appetite are more commonly associated with poor mental health after discharge for survivors of ICU than disturbance in cognitive function (Jackson et al. 2014). A strong negative association was found between MCS and presence of depression, longer duration of MV and longer ICU and hospital LOS. Longer stay in ICU and in hospital could have led to the development of the somatic symptoms described above. A limitation of this study is that the presence of somatic symptoms and its relation to mental HRQOL was not investigated.

Significant improvements were observed in physical HRQOL in this group of participants (SF-36 PCS and EQ-5D VAS). This finding might be attributed to the significant improvements in muscle strength observed during the 6 months following discharge. Most participants reported that they had some form of support from family and friends after discharge, and some of them had also returned to work by 6 months following discharge. The mean PCS score for this group of participants is lower than the mean PCS score (57.6) reported for a group of healthy South African subjects (Van Aswegen et al. 2011). This cohort had several comorbidities which may have contributed to the lower observed mean PCS scores at 6 months. Large reductions in levels of pain and discomfort experienced at 6 months, as measured with the EQ-5D questionnaire, may be because of wound healing that took place as the majority of participants underwent surgery. This might have contributed to the improvements in PCS reported for this group. Myhren et al. (2010) reported a return to work rate of 55% for their group of ICU survivors by 1 year after discharge. This is similar to the return to work rate observed in our study which was achieved by 6 months after discharge. The relatively good return to work rate for these participants may be attributed to the significant increases in muscle strength and PCS observed as well as the reduction in problems reported in relation to mobility, self-care, pain or discomfort and improved participation in usual activities over the test period. The type of occupation that participants returned to, for example office work, might also have influenced the return to work rate observed.

The study failed to meet the projected sample size. Many patients were discharged from ICU to rehabilitation or step-down centres and did not spend time in the hospital wards and therefore could not be enrolled in the study. Participants needed time to consider their willingness to participate and many went home before a final decision was made. Because of budget restrictions, it was not feasible to include more research sites in the trial. The small sample size limits the degree to which findings can be extrapolated to a larger ICU population and should therefore be interpreted with caution. Drop-out rate during the study was high (37.5% at 1 month and 54.2% at 6 months). Two longitudinal South African studies also reported high participant drop-out rates (Van Aswegen et al. 2010, 2011). Methods employed to limit drop-out rate included collection of participants’ contact details at the time of study enrolment (telephone numbers, mobile phone numbers, email addresses and physical addresses). Those who could not be reached might have changed their contact details or passed away after discharge. Visits to participants’ place of living could not be made again because of budget restrictions. Numerous attempts were made to contact all participants who did not arrive for follow-up appointments to re-schedule their appointments.

This older mixed cohort of ICU survivors with pre-existing comorbidities seemed to perform better in physical function outcomes and social outcomes than those reported for other ICU populations. Limitations in the improvement of mental HRQOL summary scores and the presence of depressive symptoms in some participants at 6 months are concerning. The lack of pre-admission information related to the various outcomes assessed for physical function and psychological health limits the extent to which post-discharge results can be interpreted as it is not known what these participants’ ‘norms’ were prior to admission. Progression of rehabilitation interventions in the form of exercise therapy, initiated during hospitalisation, in the period after discharge may be useful to further improve exercise endurance and positively impact mental health (Wegner et al. 2014). Those with a history of prolonged MV duration and hospital LOS that exhibit limitations in mental HRQOL and signs of depressive symptoms should be referred to a psychologist for evaluation.

## Conclusion

Clinically important improvements in biopsychosocial outcomes related to physical function and social factors were observed for this mixed cohort of ICU survivors. Limitations in mental aspects of HRQOL were present at 6 months and some reported mild depressive symptoms. Prolonged duration of MV and LOS has a negative impact on mental aspects of HRQOL.
